# Recent Advances in Radiofrequency Ablation for the Management of Hepatocellular Carcinoma

**DOI:** 10.5812/hepatmon.5945

**Published:** 2012-10-10

**Authors:** Takashi Himoto, Kazutaka Kurokohchi, Seishiro Watanabe, Tsutomu Masaki

**Affiliations:** 1 Department of Integrated Medicine, Kagawa, Japan; 2 Department of Gastroenterology and Neurology, School of Medicine, Kagawa University, Kagawa, Japan; 3 Department of Internal Medicine, Kagawa Prefectural Central Hospital, Kagawa, Japan

**Keywords:** Carcinoma, Hepatocellular, Treatment, Catheter Ablation

## Abstract

**Contexts:**

Hepatocellular carcinoma (HCC) is one of the most common malignant diseases in the world. Because less than 20% of patients with HCC are resectable, various types of non-surgical treatment have been developed.

**Evidence Acquisition:**

At present, radiofrequency ablation (RFA) is accepted as the standard local treatment for patients with HCC because of its superior local control and overall survival compared to other local treatments.

**Results:**

New devices for RFA and combination treatments of RFA with other procedures have been developed to improve anti-tumoral effects.

**Conclusions:**

This review mainly focuses on the status of RFA in the management of HCC and recent advances in RFA treatment technology.

## 1. Context

Hepatocellular carcinoma (HCC) is the fifth most common cancer worldwide (1), and generally arises from a precursor condition such as chronic hepatitis or liver cirrhosis. It is highly prevalent in the Asia-Pacific region and Africa (2), and is increasing in Western countries (3), with an estimated incidence ranging between 500,000 and 1,000,000 new cases annually. However, unlike in other solid tumors, surgical resection plays a limited role in the treatment of HCC. Surgery is precluded in the majority of HCC patients due to the anatomic location, size or number of tumors, or an impaired of the hepatic reserve. Only 10-20% of patients with HCC can be candidates for surgery (4). Furthermore, tumor recurrence is common, even after apparently curative resection. Liver transplantation has been carried out in well-selected patients with HCC who fulfill the Milan criteria of a solitary HCC less than 5cm or up to three nodules smaller than 3cm in diameter (5). However, the availability of liver transplantation is extremely restricted by the shortage of organ donors. Because of the circumstances described above, various types of non-surgical treatments have been introduced. Transarterial chemoembolization (TACE) using various anti-cancer agents (doxorubicin, mitomycin, and cisplatin) and embolizing agents (geratin and microspheres) has been well documented (6). On the other hand, ultrasound-guided locoregional treatments have also been developed, as an alternative to surgery, in patients with HCC. Tumor ablation can be achieved by modifying the temperature of tumor cells (microwave (7), laser, cryoablation (8), and radiofrequency (9) or by injecting chemical substance including ethanol (10) and acetic acid (11) into the tumor nodules. At present, radiofrequency ablation (RFA) is well established as the standard local treatment for HCC because of its superior rates of local control, overall survival, and cancer-free survival compared to other local treatments (12-16).

Recently, molecular targeted systemic therapy with sorafenib (17) has been introduced in patients with HCC. Sorafenib, a multikinase inhibitor with antiangiogenic properties, has been shown to prolong median overall survival compared to placebo in a randomized control study. This article mainly focuses on present status of RFA in the management of HCC and recent advances in RFA treatment technology.

## 2. Evidence Acquisition

### 2.1. Indications for RFA

**Figure 1:**
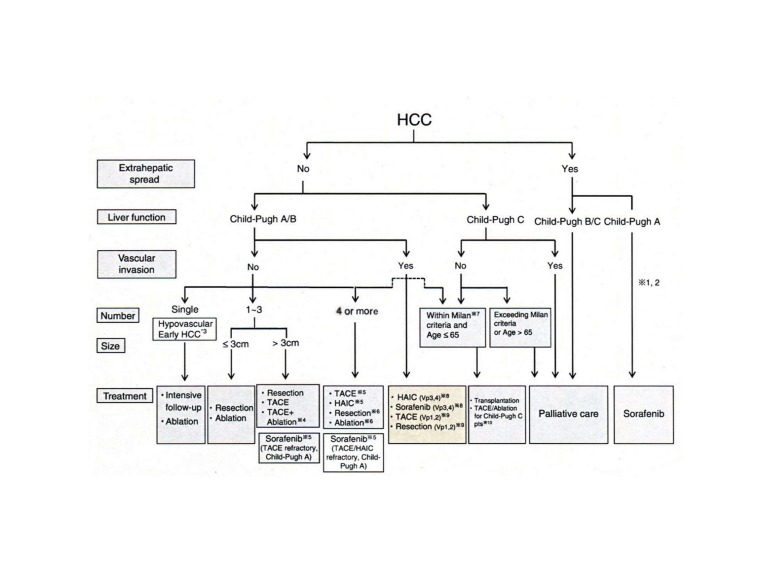
Treatment Algorithm for HCC Proposed by Japanese Society of Hepatology in 2010 Cited From the Reference Reported by Arii et al. (21)

Percutaneous ethanol injection (PEI), the injection of ethanol directly into the tumor through a fine needle under the guidance of ultrasonograpghy, was initially developed in Japan as a local treatment for HCC in the early 1980s (10). Intratumoral injection of ethanol leads to nonselective protein degradation and cellular dehydration, resulting in coagulative necrosis within the tumor. Some years later, OK-432, a streptococcal preparation which induces multiple cytokines for anti-cancer effects (18, 19), and acetic acid (11) were applied as additional substances to locally injected into the tumors. Then, in the late 1990s, microcoagulation therapy (MCT) became more common in Japan. MCT ablates the tumors by producing dielectric heat emitted from an inserted electrode. Now, RFA is considered the most promising procedure as a locoregional treatment for HCC. This procedure leads to coagulative necrosis and tissue desiccation by delivering high-frequency alternating current via electrodes placed within the tissues. RFA seems to be superior to PEI in all tumor sizes of HCC due to its stronger necrotic effects (20). MCT has been mostly replaced with RFA due to difficulty in controlling the ablation power by microcoagulation. Recently, an algorithm of HCC treatment has been proposed by the Japanese Society of Hepatology (Figure 1) (21). According to the algorithm, the treatment of HCC depends on liver damage, the number of tumors, tumor size, and the presence or absence of distant metastasis. Currently, three or fewer tumors with a diameter of 3cm or smaller and no extrahepatic lesions, well-preserved liver function, and no vascular invasions, are generally indications for RFA (22).

### 2.2. Comparisons of the Outcomes between RFA and Other Treatments

There are several randomized control trials comparing RFA with PEI for the management of HCC (23-31), as shown in Table 1. The numbers of treatment sessions, complete therapeutic effect, overall survivals, and rate of severe complication were compared between RFA and PEI groups in these articles. RFA resulted in a higher rate of complete necrosis than PEI, although no significant difference was apparent, and required significantly fewer treatment sessions than PEI. However, a meta-analysis revealed that RFA was not significantly better than PEI for tumors ≤ 2cm (32). The better local control by RFA in comparison with PEI seemed to derive from the stronger and more expansive coagulative effects of thermal ablation on the HCC nodules and micro-satellites around the tumors. The homogeneous distribution of injected ethanol is largely disturbed by interference from the intratumoral fibrotic septum or the presence of satellite nodules around the target tumors (33). In contrast, heat generated around the radiofrequency electrode tip is usually distributed quite homogeneously in all directions. Therefore, RFA frequently makes stronger ablation possible. The survival rate indicated a significant benefit for RFA over PEI; the more favorable survival may derive from the higher rate of complete response in RFA than in PEI, because an initial complete response is an independent predictor of survival (34). However, the rate of major complications was higher with RFA than with PEI, although the difference was not statistically significant. Therefore, we should consider the locoregional treatment as part of the overall risk/benefit profile in each individual. There have been a few randomized control studies comparing RFA with previously reported MCT (35-38). These studies estimated that RFA has an almost similar or slightly superior effect on the local tumor control rates and survivals compared to MCT. However, the coagulated area produced by MCT is usually smaller than those produced by RFA; thus MCT requires more sessions to obtain complete therapeutic effects in comparison with RFA. There have also been several randomized and non-randomized control studies comparing RFA with hepatic resection (HR) (39-49) (Table 2). Zhou and colleagues performed a meta-analysis of these articles to assess the efficacy of RFA and HR for the treatment of HCC (50). According to their analysis, the overall survival was significantly higher in patients treated with HR than in those treated with RFA at 3 years. On the other hand, RFA showed a significantly higher rate of local intrahepatic recurrence, compared to HR. However, a few non-randomized control trials revealed that RFA did not differ significantly from HR for survival in tumors equal to or less than 3 cm in diameter.

**Table 1:** Summary of the Comparative Studies on RFA vs. PEI in Patients with Hepatocellular Carcinoma

	Inclusion Criteria	Nodules, No.	Treatment Session Per Nodule	*P *value	CompleteTherapeutic Effect a, %	*P * value	OverallSurvival, %	*P *value	Rate of MajorComplications, %	*P *value
**Livraghi *et al.*(1999) (23)**	nodule < 3cm	RFA (n = 52), PEI(n = 60)	1.2 *vs.*4.8	-	90 *vs*. 80	NS	notdescribed	-	10 *vs. *0	NS
**Ikeda *et al. *(2001) (24)**	nodule < 3 cm	RFA (n = 23), PEI(n = 96)	1.5 *vs. *4.0	*P* <0.01	100 *vs. *94	NS	notdescribed	-	0 *vs. *1	NS
**Lencioni *et al. *(2003) (25)**	single tumor < 5 cm and nodule < 3 cmand < 3 nodules	RFA (n = 52), PEI(n = 50)	1.1 *vs. *5.4	-	91 *vs. *82	-	98 *vs. *88 (2 y)	NS	not described	-
**Lin *et al. *(2004) (26)**	< 4 cm of nodule	RFA (n = 52),RFA (n = 52)	not described		96 *vs. *88	NS	74 *vs. *50 (3 y)	*P *=0.0017	2 *vs. *0	NS
**Lin *et al. *(2005) (27)**	nodule < 3 cm and <3 nodules	RFA (n = 75), PEI(n = 67)	1.3 *vs. *4.9	*P *<0.01	96 *vs. *88	NS	74 *vs. *51 (3 y)	*P *=0.031	5 *vs. *0	*P*=0.035
**Shiina *et al. *(2005) (28)**	nodule < 3 cm and <3 nodules	RFA (n = 118), PEI(n = 114)	2.1 *vs. *6.4	-	100 *vs. *100	-	74 *vs. *57 (4 y)	NS	5 *vs. *3	NS
**Luo *et al.*(2005) (29)**	nodule < 3 cm and <3 nodules	RFA (n = 153), PEI(n = 85)	not described	-	92 *vs.*78	NS	64 *vs. *53 (3 y)	NS	not described	
**Seror *et al. *(2006) (30)**	nodules < 3.5 cm and child-Pugh Acirrhosis	RFA (n = 72), PEI(n = 72)	1.1 *vs. *4.3	NS	99 *vs. *71	*P *=0.0001	91 *vs. *71 (2 y)	*P *=0.006	15 *vs. *7	NS
**Brunello *et al. *(2008) (31)**	nodule < 3 cm and< 3 nodules or child-Pugh A /B cirrhosis	RFA (n = 70), PEI(n = 69)	not described	-	96 *vs. *66	*P*=0.0001	59 *vs. *57 (3 y)	NS	3 *vs. *3	NS

Abbreviations: NS, not significant; PEI, Percutaneous ethanol injection; RFA, radiofrequency ablation.

^a^RFA vs. PEI

**Table 2:** Summary of the Comparative Studies on RFA vs. Hepatic Resection in Patients with Hepatocellular Carcinoma

	Inclusion Criteria	Patients, No.	OverallSurvival a, 3 y, %	*P* value	IntrahepaticRecurrence a, %	*P* value	Complication, %	*P* value
**Vivarelli *et al.*(2004) (38)**	Child A/B liver cirrhosis	RFA (n = 79), Resection(n = 79)	33 *vs. *65	*P* = 0.002	33 *vs. *65		0 *vs. *4	NS
**Hong *et al.*(2005) (39)**	one nodule < 4 cm and child A livercirrhosis	RFA (n = 55), Resection(n = 93)	73 *vs. *84	NS	40 *vs. *43	NS	not described	
**Montorsi *et al.*(2005) (40)**	one nodule < 5 cm and child A/B livercirrhosis	RFA (n = 58), Resection(n = 40)	45 *vs. *61 (4 y)		53 *vs*. 30	*P *=0.018	not described	
**Cho *et al.*(2005) (41)**	nodules < 5 cm and < 3 nodules	RFA (n = 99), Resection(n = 61)	80 *vs. *77	NS	18 *vs. *10	NS	5 *vs. *7	NS
	Child A liver cirrhosis							
**Ogihara *et al.*(2005) (42)**	not described	RFA (n = 40), Resection(n = 47)	58 *vs. *65	NS	25 *vs*. 28	NS	not described	
** Lu *et al. *(2006)(43)**	not described	RFA (n = 51), Resection(n = 54)	87 *vs. *86	NS	28 17	NS	8 *vs. *11	NS
**Chen *et al.*(2006) (44)**	one nodule < 5 cm and child A livercirrhosis	RFA (n = 71), Resection(n = 90)	69 *vs. *73	NS	not described		4 *vs. *56	*P* < 0.05
**Lupo *et al.*(2007) (45)**	one nodule < 5 cm and Child A livercirrhosis	RFA (n = 60), Resection(n = 42)	53 *vs. *57	NS	not described		10 *vs*. 17	NS
**Hasegawa *et al.*(2008) (46)**	nodules < 3 cm and < 3 nodules and ChildA/B liver cirrhosis	RFA (n = 3022),Resection (n = 2857)	93 *vs. *95 (2 y)		26 *vs. *17	NS	not described	
**Gugliemi *et al.*(2008) (47)**	nodules < 6 cm	RFA (109), Resection(n = 91)	42 *vs. *64	*P* = 0.01	not described		not described	
**Abu-Hilal *et al.*(2008) (48)**	one nodule < 5 cm	RFA (n = 32), Resection(n = 32)	81 *vs. *63 (2 y)	NS	not described		not described	
**Ueno S *et al.*(2009) (49)**	one nodule < 5 cm or nodules < 3 cm and< 3 nodules	RFA (n = 155), Resection(n = 123)	92 *vs. *92	NS	not described		not described	

Abbreviations: NS, not significant; PEI, Percutaneous ethanol injection; RFA, radiofrequency ablation.

^a^RFA vs. Resection.

### 2.3. Limitations and Pitfalls of RFA

As described above, RFA has many favorable effects on the treatment for HCC. However, there are several limitations and pitfalls of the treatment with RFA, including limited ablation volume, location of HCC, heat sink effect, and neoplastic seeding. The ablation zone by the currently available RFA technology is limited up to 4-5 cm in maximum diameter (14). On the other hand, the treatment for HCC tumors in subcapsular location or adjacent to the gall-bladder increased the risk of incomplete ablation (16). The presence of large vessels close to the tumors also has the negative effect on thermal ablation, which is called “heat sink effect” (51). Moreover, neoplastic seeding is well known as one of complications of RFA technique (15).

### 2.4. Modified Techniques of RFA

RFA for HCC is mainly accomplished by a percutaneous approach, although open (52), laparoscopic (53, 54), or thoracoscopic approaches (55) can also be used. In the previous study, the injection of 5% glucose solution into the intrapleural cavity as an artificial pleural effusion enabled us to detect tumors located in subdiaphragm and to treat them very successfully with RFA (56). Recently, real-time virtual sonography (RVS)-guided RFA was introduced for using in tumors that are unclear on B-mode ultrasonography (57). This technique drastically increased the therapeutic efficacy. Also, carbon dioxide microbubbles (58) and sonazoid-enhanced ultrasonography (59, 60) are useful procedures for detection of unclear tumors on B-mode ultrasonography. To enhance anti-tumor effects through RFA, several kinds of techniques have been designed. We developed a combination therapy using RFA and PEI (PEI-RFA) for the treatment of HCC nodules (29, 53, 61-67). Our study using bovine livers confirmed that the coagulation by this combination treatment was more expansive than that by RFA alone (62). Yamasaki and colleagues successfully performed RFA combined with hepatic arterial balloon occlusion for larger tumors (68). There is controversy about the efficacy of RFA in HCCs exceeding 3cm in diameter. Recent studies have focused on the combination treatment using TACE and RFA against large HCCs (69-71). For such huge tumors, lipiodol TACE-proceded RFA is widely performed, with the aim at the treating satellite nodules and microscopic vascular invasion and ensuring an accurate margin by lipiodol injection. Lipiodol TACE-preceded RFA is relatively curative and shows a favorable survival almost equivalent to HR (72).

## 3. Results

The therapy of RFA and subsequent administration of an active antigen-specific immunotherapeutic approach using dendritic cells (73) may be an appropriate option for the enhancement of antitumoral effects, reducing tumor recurrence and metastasis in patients with HCC. The combination treatment of RFA and targeted systemic therapy including sorafenib may also be a novel option for the improvement of treatment outcome.

## 4. Conclusions

RFA has become the standard local treatment against HCC because of its more favorable survival and local disease control compared to other local treatments. RFA should be considered as a first-line treatment for small HCCs (equal to or less than 3cm in diameter). RFA treatment is as effective as HR for the treatment of HCCs equal to or less than 3cm with respect to overall survival. Combination therapy of RFA and PEI, or TACE is performed in large tumors for enhancement of antitumoral effects.
